# Nucleic Acids Detection for *Mycobacterium tuberculosis* Based on Gold Nanoparticles Counting and Rolling-Circle Amplification

**DOI:** 10.3390/bios12070448

**Published:** 2022-06-23

**Authors:** Xiaojing Pei, Hu Hong, Sitong Liu, Na Li

**Affiliations:** 1 Institute of Cosmetic Regulatory Science, College of Chemistry and Materials Engineering, Beijing Technology and Business University, Beijing 100048, China; pxj@btbu.edu.cn (X.P.); 2130041001@st.btbu.edu.cn (S.L.); 2 Beijing National Laboratory for Molecular Sciences (BNLMS), Key Laboratory of Bioorganic Chemistry and Molecular Engineering of Ministry of Education, Institute of Analytical Chemistry, College of Chemistry and Molecular Engineering, Peking University, Beijing 100871, China; eeeqxxtg@pku.edu.cn

**Keywords:** tuberculosis, nucleic acids, gold nanoparticles counting, rolling-circle amplification

## Abstract

Tuberculosis (TB) is a common infectious disease caused by *Mycobacterium tuberculosis*, which usually disturbs the lungs, and remains the second leading cause of death from an infectious disease worldwide after the human immunodeficiency virus. Herein, we constructed a simple and sensitive method for *Mycobacterium tuberculosis*-specific DNA detection with the dark-field microscopic imaging of gold nanoparticles (AuNPs) counting strategy and rolling-circle amplification (RCA). Taking advantage of RCA amplification, one target molecule produced hundreds of general oligonucleotides, which could form the sandwich structure with capture-strand-modified magnetic beads and AuNPs. After magnetic separation, AuNPs were released and detected by dark-field imaging; about 10 fM *Mycobacterium tuberculosis*-specific DNA target can still be differentiated from the blank. No significant change of the absorbance signals was observed when the target DNA to genomic DNA ratio (in mass) was from 1:0 to 1:10^6^. The spike recovery results in genomic DNA from human and *Klebsiella pneumoniae* suggested that the proposed method has the feasibility for application with biological samples. This proposed method is performed on an entry-level dark-field microscope setup with only a 6 μL detection volume, which creates a new, simple, sensitive, and valuable tool for pathogen detection.

## 1. Introduction

Tuberculosis (TB) has long been a crucial global health issue and is still one of the top 10 causes of death worldwide, which is caused by acid-fast *Mycobacterium tuberculosis* (Mtb) [1,2,3,4]. Early diagnosis and treatment for tuberculosis were essential for effectively controlling pandemics. Conventional TB diagnostics include the tuberculin test and sputum smear microscopy, which are evaluated to have several problems including poor sensitivity, specificity, and false negatives [5]. The interferon-γ release assay has shown promise with fitting sensitivity, but it cannot differentiate between active TB disease and latent infection [6]. Mtb culture was taken as the gold standard for diagnosis. However, it is a lengthy process increasing the risk of continued transmission [7]. Nucleic acids, including deoxyribonucleic acid (DNA) and ribonucleic acid (RNA), have been considered specific biomarkers for clinical diagnostics [8,9,10]. Most available diagnostic methods use polymerase chain reaction (PCR) or sequencing-based technologies to detect nucleic acid-specific to Mtb [11,12,13]. However, these methods require sophisticated laboratory instruments and complicating procedures, which is unaffordable for resource-limited settings.

Gold nanoparticles (AuNPs) were widely used in ingenious biosensor designs and accurate clinical diagnoses [11,14,15,16,17]. In addition to the simplicity of preparation and modification, AuNPs present a substantial molecular extinction cross-section, resonance Rayleigh scattering efficiency, and enhanced local electromagnetic field because of localized surface plasmon resonance (LSPR) [18,19,20,21]. It is reported that the optical cross-sections of AuNPs with a size of 10–100 nm are about orders of magnitude higher than those of common fluorescent dyes [22]. Accordingly, via the specific recognition event, the invisible target molecules can be quantitatively by counting the number of AuNPs with the dark-field microscope (DFM). Due to the necessity for sophisticated instrumentation and nanoparticle monodispersity being significantly reduced, the analytical methods based on AuNPs single-nanoparticle counting method show promising potential for achieving high sensitivity in practical applications [23,24,25,26,27]. Many fantastic efforts have been made using AuNPs as signal sources. For instance, Li and co-workers developed an automatic particle counting approach at the single-particle level, which serves as a general and straightforward sensing platform [28,29]. However, the sensitivity is not satisfactory; moreover, there is a requirement for well-trained personnel operations. Furthermore, the possibility of combining amplification techniques with AuNP counting has not been explored systematically. Rolling circle amplification (RCA) involves isothermal DNA replication and is a frequently used technique that combines simplicity with sensitivity [30]. Trace targets will produce high-molecular-weight linear products by RCA reaction [31,32]. Herein, we constructed an ultra-sensitive method for *Mycobacterium tuberculosis*-specific DNA with the dark-field microscopic imaging of AuNPs counting strategy after RCA reactions. Taking advantage of RCA, every single target molecule produces a number of general oligonucleotides, which could form the sandwich structure with capture-strand-modified magnetic beads and AuNPs. After magnetic separation, AuNPs were released and detected by dark-field imaging, showing promising potential applications in clinical diagnosis and pathogen detection.

## 2. Materials and Methods

***Chemicals and materials.*** All synthetic DNA oligonucleotides in Table S1 were purchased from Sangon Biotech Co., Ltd. (Shanghai, China). Auric chloride dehydrates of A. R. grade and Triton X-100 (C.P.) were purchased from Sinopharm Chemical Reagent Co., Ltd. (Beijing, China). K_2_HPO_4_, NaH_2_PO_4_, NaCl, NaOH, HAuCl_4_, EtOH, and MeOH, all of A. R. grade, were obtained from Beijing Chemical Works (Beijing, China). Streptavidin-modified magnetic beads (Dynabeads MyOne Streptavidin T1, *d* = 1 μm, 10 mg/mL) were purchased from Thermo Fisher Scientific (Waltham, MA, USA). Coverslips are obtained from Shitai Co., Ltd. (Jiangsu, China). Glass slides are obtained from Matsunami Glass Ind., Ltd. Tris (2-carboxyethyl) phosphine (TCEP) and bovine serum albumin (BSA) are obtained from Sangon Biotech Co., Ltd. (Shanghai, China). The calf thymus was purchased from Solarbio Life science. The Nucleic Acid Kit was purchased from Roche and Solarbio Life science as used for Nucleic Acid extraction from healthy donors and *klebsiella pneumonia.* Phi29 DNA polymerase and BtsCI Restriction Endonuclease were obtained from New England Biolabs (Beijing, China) LTD. T4 DNA ligase, dNTPs, exonuclease I, and exonuclease III are supplied by TaKaRa Biotechnology (Dalian, China) Co.,Ltd.

***Apparatus.*** The images were taken using an Olympus BX-53 microscope with a Olympus DP-72 genuine color CCD. The image processing software was developed in the C^#^ programming language, based on the work of Li’s group [33]. The characterization of AuNPs with shape and size is taken by JEM2100 transmission electron microscopy (TEM).

***Synthesis of Gold Nanoparticles.*** The AuNPs were synthesized by kinetically controlled seeded growth methods reported in the literature [34]. Specifically, a solution of 2.2 mM sodium citrate in 150 mL water was heated for 15 min under vigorous stirring. After boiling, 1 mL of 25 mM HAuCl_4_ solution was injected. The color of the solution changed to yellow, bluish–gray, and soft pink gradually, and the particles were about 10 nm. Then, the mixture was cooled to 90 °C. Then, 1 mL of 25 mM HAuCl_4_ solution and 1 mL of 60 mM sodium citrate were sequentially injected within 2 min. By repeating this procedure, up to 6 generations of AuNPs of progressively larger sizes were produced. 

***Functionalization of AuNPs.*** AuNPs functionalization was carried out at 25 °C based on the salt aging method [35]. Specifically, 100 μL of 10 μM Signaling DNA was transferred into a microcentrifuge tube, and 10 μL of 10 mM TCEP was added to activate the thiol-modified Signaling DNA. After incubation at 25 °C for 1 h, 1 mL of the above prepared gold nanoparticles was added to the TCEP-treated Signaling DNA, and the resultant mixture was stored at 4 ℃ for 16 h. Subsequently, 100 μL of 50 mM PBS (pH 7.6) was added with gentle shaking. The resultant phosphate concentration was 5 mM. Then, 100 μL of 1 M NaCl was added with gentle vibration. The reaction solution was stored in the dark for 24 h. Excess Signaling DNA was then removed by centrifugation for 7 min at 4500 rpm. Following removal of the supernatant, the red precipitate was washed three times with deionized water and then finally redispersed in fresh PBS.

***Mycobacterium tuberculosis DNA Detection.*** 10 μL 10× T4 reaction buffer, 10 μL 10× phi29 reaction buffer, and 30 μL 50 nM circle template were mixed as template solution. Then, 5 μL of the template solution above and 15 μL target were transferred into a microcentrifuge tube. The solution was cooled from 75 °C to 25 °C. Then, 100 U T4 ligase was added and incubated at 16 °C for 30 min; 10 U Exonuclease I was added and kept at 37 °C for 1 h. Afterward, the mixture was heated at 80 °C for 15 min to inactivate it, and then it was cooled to 30 °C. Subsequently, 2 μL dNTP, 0.2 μL of 10 mg/mL BSA and 5U Phi29 DNA polymerase was added and kept at 30 °C for 2 h, and heat-denaturalization was performed at 65 °C for 10 min. Then, 60 μL enzyme digestion solution was added. Each 100 μL of enzyme digestion solution contained 10 μL of 10× CutSmart buffer and 1 μL 20 U BtsGI restriction endonuclease. The mixture was kept at 50 °C for 1 h, and heat-denaturalization was performed at at 80 °C for 15 min. Then, 100 μL of 200 nM capture DNA was added and annealed from 75 °C to 25 °C; 10 μL of 10 mg/mL streptavidin modified magnetic beads was added later and kept at 1200 rpm for 20 min at 25 °C. After magnetic separation, the mixture was washed with PBS with 0.1% Triton X-100 2 times. Then, 30 μL of Signaling DNA labeled AuNPs was added and incubated at 48 °C for 5 min. Then, the mixture was slowly annealed to 25 °C within 15 min. After magnetic separation, the mixture was washed 4 times with PBS containing 0.1% Triton X-100 and 1 time with PBS without surfactant. Finally, 20 μL of 0.15 M NaOH was added to de-hybridization and AuNPs were released. Ten minutes later, 6 μL supernatant and 6 μL of 0.5 M KH_2_PO_4_ solution were mixed and applied to the glass slide, the glass slide was washed for 10 min with deionized water, and the dark-field image was obtained with the DFM.

***Detection of target DNA in the presence of genomic DNA.*** The assay was carried out in the presence of a large excess of Calf thymus DNA (ctDNA), which was used as an imitator for a complicated matrix. Target DNA/ctDNA weight ratios varied from 1:0 to 1:10^6^. The concentration of target DNA was 1 pM and the detection procedures were identical to same procedure described above.

***Spike Recovery of target from genomic DNAs of human and Klebsiella pneumoniae.*** Twenty oropharyngeal swab samples were acquired from healthy donors in our laboratory. The genomic DNAs from oropharyngeal swab samples were extracted from using a commercial kit from Roche according to the manufacturer’s instructions. The extracted genomic DNAs were eluted with elution buffer, and the final volume was 900 μL. All *Klebsiella pneumoniae* strains in our work were provided by (>10^8^ CFU/mL) was provided by Tong laboratory (Beijing university of chemical technology) and stored at −80 °C in 30% (*v*/*v*) glycerol and cultivated at 37 °C with shaking at 220 rpm in Luria–Bertani (LB) medium for 10 h. The genomic DNAs from *Klebsiella pneumoniae* were extracted from using a commercial kit from Solarbio Life science according to the manufacturer’s instructions. The extracted genomic DNAs were eluted with elution buffer, and the final volume was 300 μL. A volume of 15 μL of target DNA with concentrations as indicated and 15 μL of extracted genomic DNAs were added in the assay mixture with a final volume of 40 μL. Assays without the standard targets were carried out in parallel. Then, quantification was carried by following the same procedure described above, and spike recovery was calculated.

## 3. Results and Discussion

**Scheme 1:**
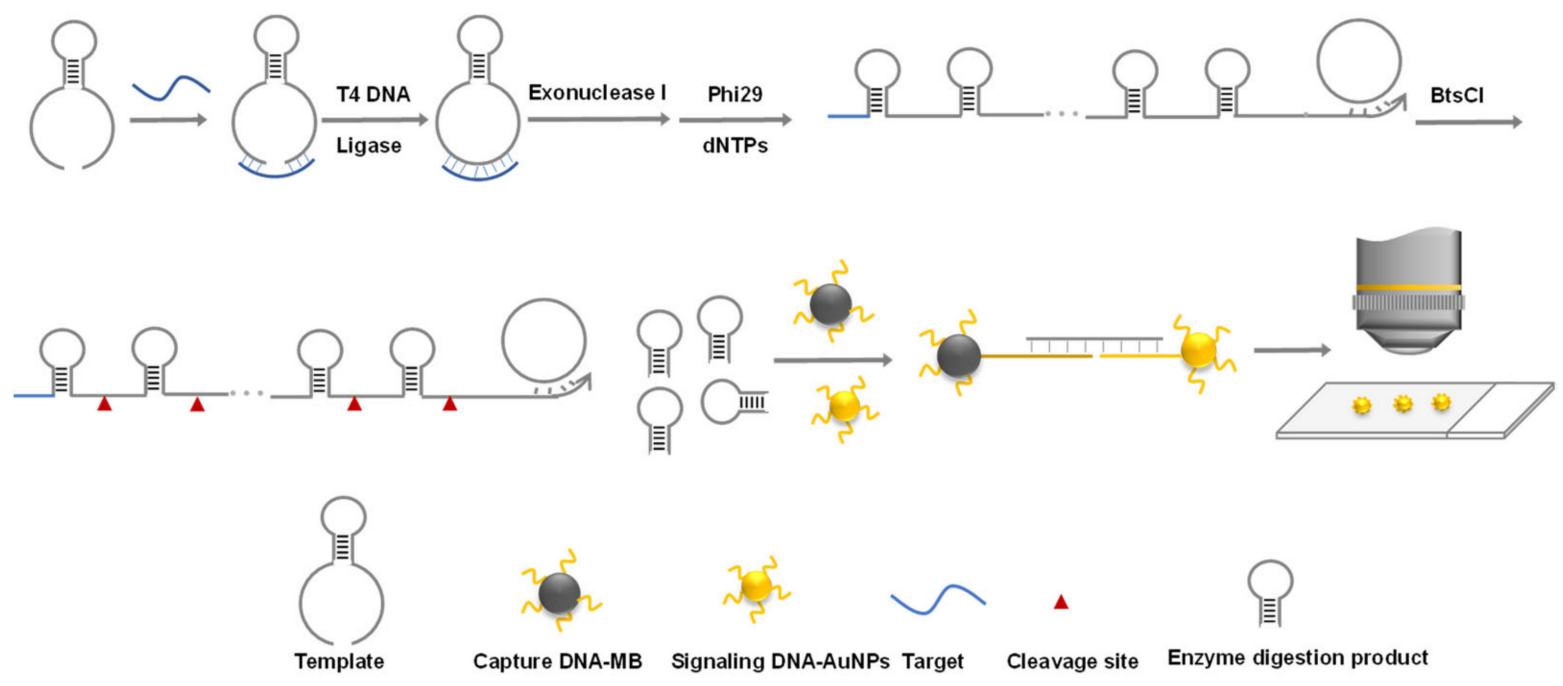
Schematic illustration of nucleic acids detection for *Mycobacterium tuberculosis* based on gold nanoparticle counting and rolling-circle amplification.

*Mycobacterium tuberculosis* nucleic acid detection based on single-nanoparticle counting and rolling-circle amplification was exemplified in Scheme 1. One target molecule produced a number of oligonucleotides after RCA and restriction enzyme digestion. The resultant oligonucleotides hybridized with the capture DNA of AuNPs and magnetic beads, forming a sandwich structure. After magnetic separation, 0.15 M NaOH was added to de-hybridization and released AuNPs from the magnetic beads’ surface. The obtained AuNPs supernatant are applied onto the amine-modified coverslip, on which the negatively charged AuNPs can be evenly distributed to facilitate high-quality image acquisition. The amount of target DNA was determined quantitatively by counting the number of AuNPs automatically in the dark-field image by C^#^-program recognition. Specifically, a dumbbell ring template with a restriction site was designed for T4 ligation. The template is universal for various targets except for the sequence related to the target. Thus, the capture DNA sequence on magnetic beads and AuNPs needs no changes with different targets, which reduces costs significantly. This shed light on the development of the AuNPs biosensor for multiple targets from other genes with remarkably reduced cost. The excessive templates will be cut down with DNA exonuclease I. Then, the RCA reaction was started with Phi29 DNA polymerase and dNTPs. Subsequently, RCA products were digested by BtsCI restriction endonuclease, producing mant short oligonucleotides. After the RCA reaction, DNA-HS AuNPs and capture DNA-labeled magnetic beads were added to form the sandwich structure for creating more effective signals.

**Figure 1:**
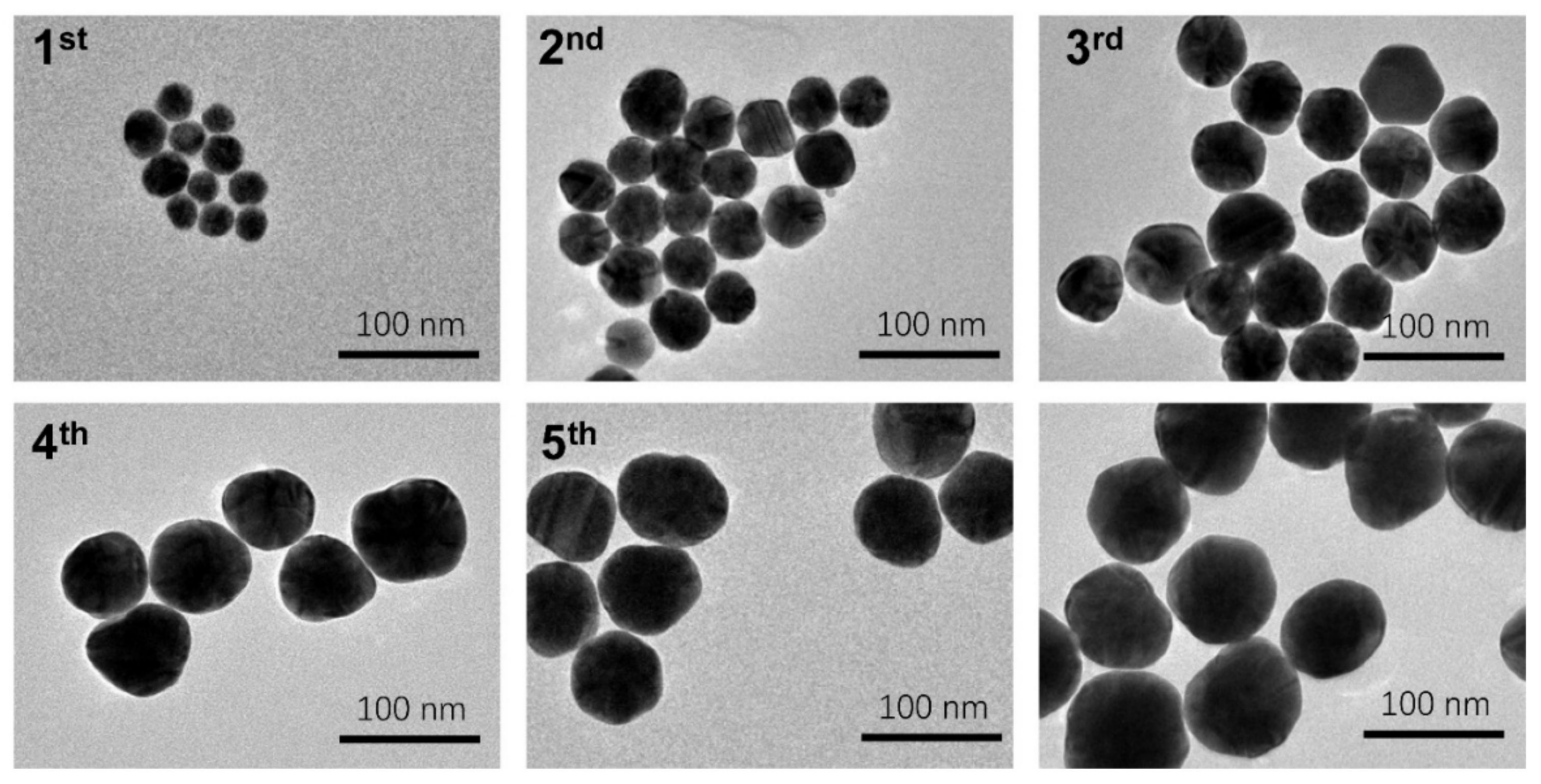
TEM images of AuNPs with different growth steps.

**Figure 2:**
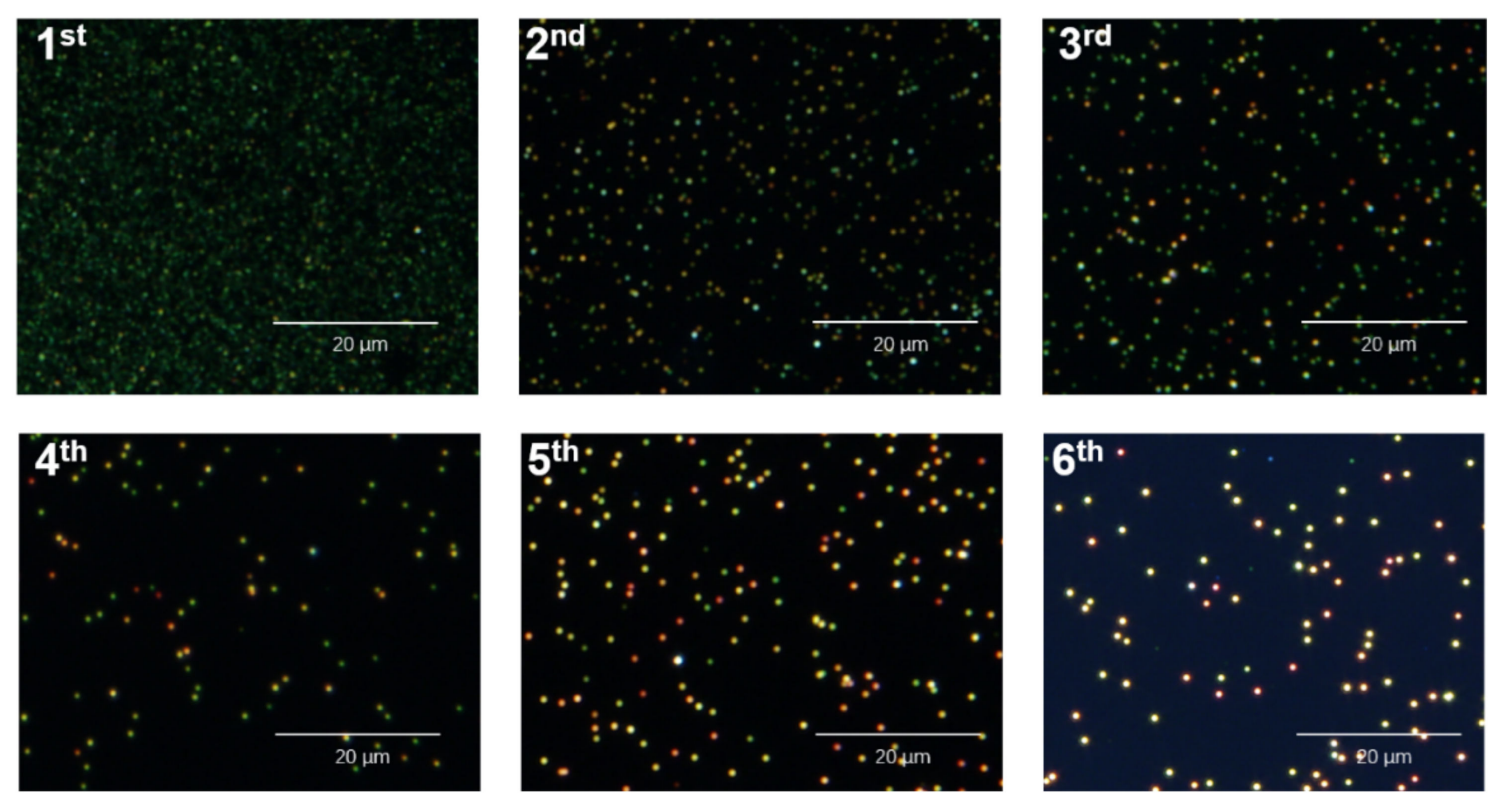
Dark-field images of AuNPs with different growth steps.

In this work, different sizes of AuNPs were synthesized by kinetically controlled seeded growth synthesis methods [34]. The size, morphology, and concentration of AuNPs were adjusted by both the number of gold atoms and the seed particle concentration added into the solution. The resultant AuNPs were characterized by transmission electron microscopic images. As shown in Figure 1, the size of the Au NPs increased with growth steps, the average sizes of the first to sixth growth steps are 25 ± 4 nm, 39 ± 5 nm, 50 ± 4 nm, 65 ± 6 nm, 68 ± 4 nm, and 79 ± 8 nm, respectively. The corresponding dark-field images are shown in Figure 2. Negatively charged AuNPs were evenly distributed onto the surface of the amino-modified coverslip. In all cases, Au NPs can be visualized clearly as bright spots in the dark-field images with the characterized plasmonic color due to the large optical absorption and scattering cross-section, which can be effectively counted by using dark-field imaging and C^#^-program recognition [33]. The brightness of AuNPs in the image was improved obviously with increased particle size. In particular, the size of AuNPs less than 40 nm exhibits a uniform green color, and they turn yellow or red with a larger particle size. Yet it did not compromise the recognition and enumeration of the C^#^ program [33]. As there is no necessity for high-level instrumentation and nanoparticle monodispersity, the analytical methods based on AuNP single-nanoparticle counting approach have shown great potential for achieving high sensitivity easily in practical applications. Considering the brightness of AuNPs spots, we chose 60 nm AuNPs of the fifth growth step as a signal source for dark-field imaging for nanoparticle counting. Due to the concentration of targets being highly associated with the number of AuNPs in dark-field images, the amount of target DNA can, thus, be calibrated and quantified by counting the number of AuNPs.

To achieve high sensitivity and specificity of nucleic acids detection for *Mycobacterium tuberculosis*, we evaluated some major factors that may influence analytical performance. Firstly, we explored DNA-HS density on AuNPs’ surface. The number of DNA capture strands attached to the surface of AuNPs can affect the stability of AuNPs and the hybridization efficiency of the probes. To obtain more effective signals, we investigated three groups of DNA-HS concentration as 0.1 μM, 0.25 μM, and 1 μM on a certain amount AuNPs. As shown in Figure S1a, the number of bright spots increased with higher DNA-HS concentration, and 1.0 μM DNA-HS and 41 pM AuNPs were determined for the following experiment. In addition, considering the balance of reaction time and efficiency, we explored the reaction time of RCA, and 1 h was optimized for the high signal; longer response times are unhelpful for signal enhancement (Figure S1b**)**. Furthermore, we evaluated the hybridization temperature of the RCA products with AuNPs. It was found that 51 °C is the optimal condition. If the temperature is too low, hybridization efficiency decreases, and in turn, non-specific adsorption increases (Figure S1c).

**Figure 3:**
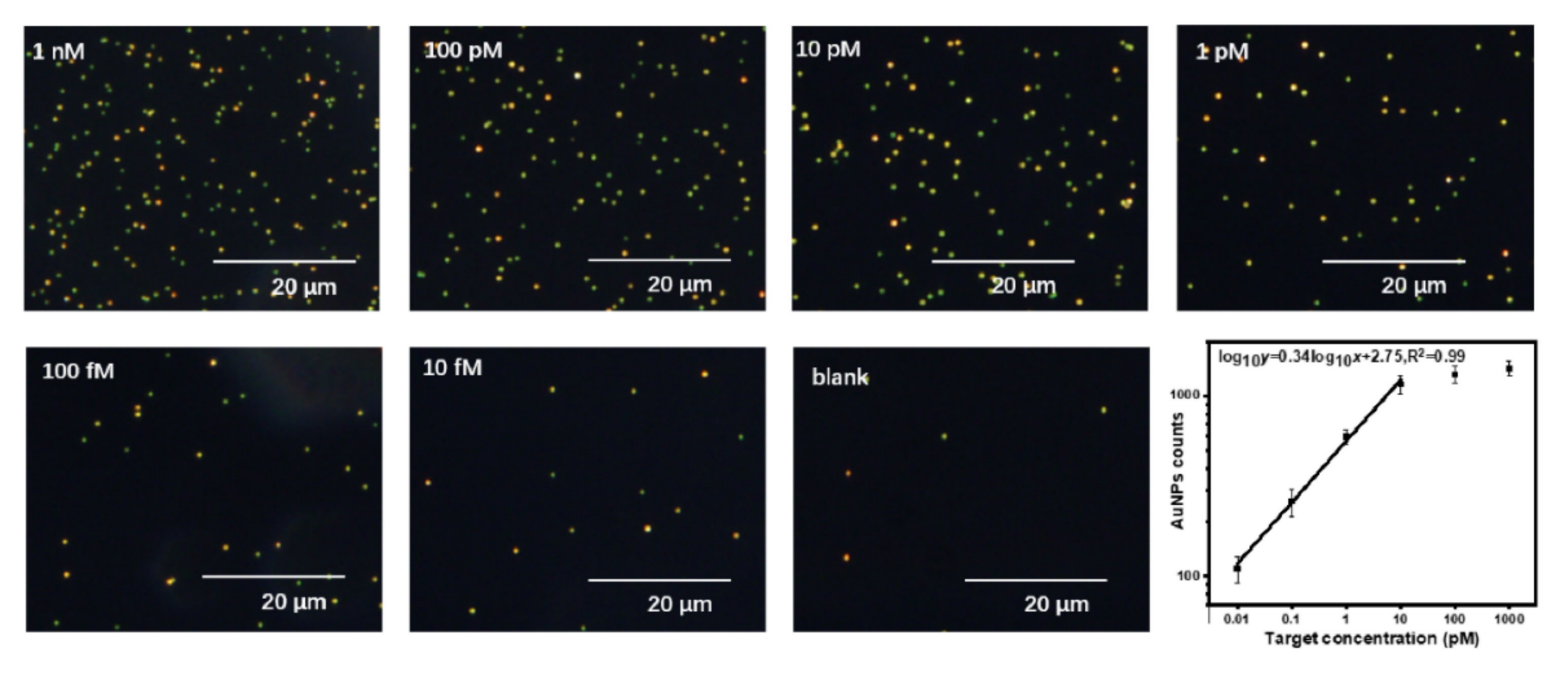
Dark-field images of Au nanoparticles and those obtained after different growth steps.

With optimized experimental conditions, the target concentrations ranging from 10 fM to 10 pM were able to be quantified with linear fit formation log_10_y = 0.34 log_10_x + 2.75, *R*^2^ = 0.99. The calibration curve presents a wide dynamic range with a detection limit of 10 fM that could still be distinguished from the blank (Figure 3). Compared to the published works based on gold nanomaterials for Mycobacterium Tuberculosis detection, the approximate10 fM sensitivity achieved with the proposed method is by far among the highest level (Table S2), showing great potential the potential to be applied to clinical cases where patients might be in an early stage of tuberculosis.

**Figure 4:**
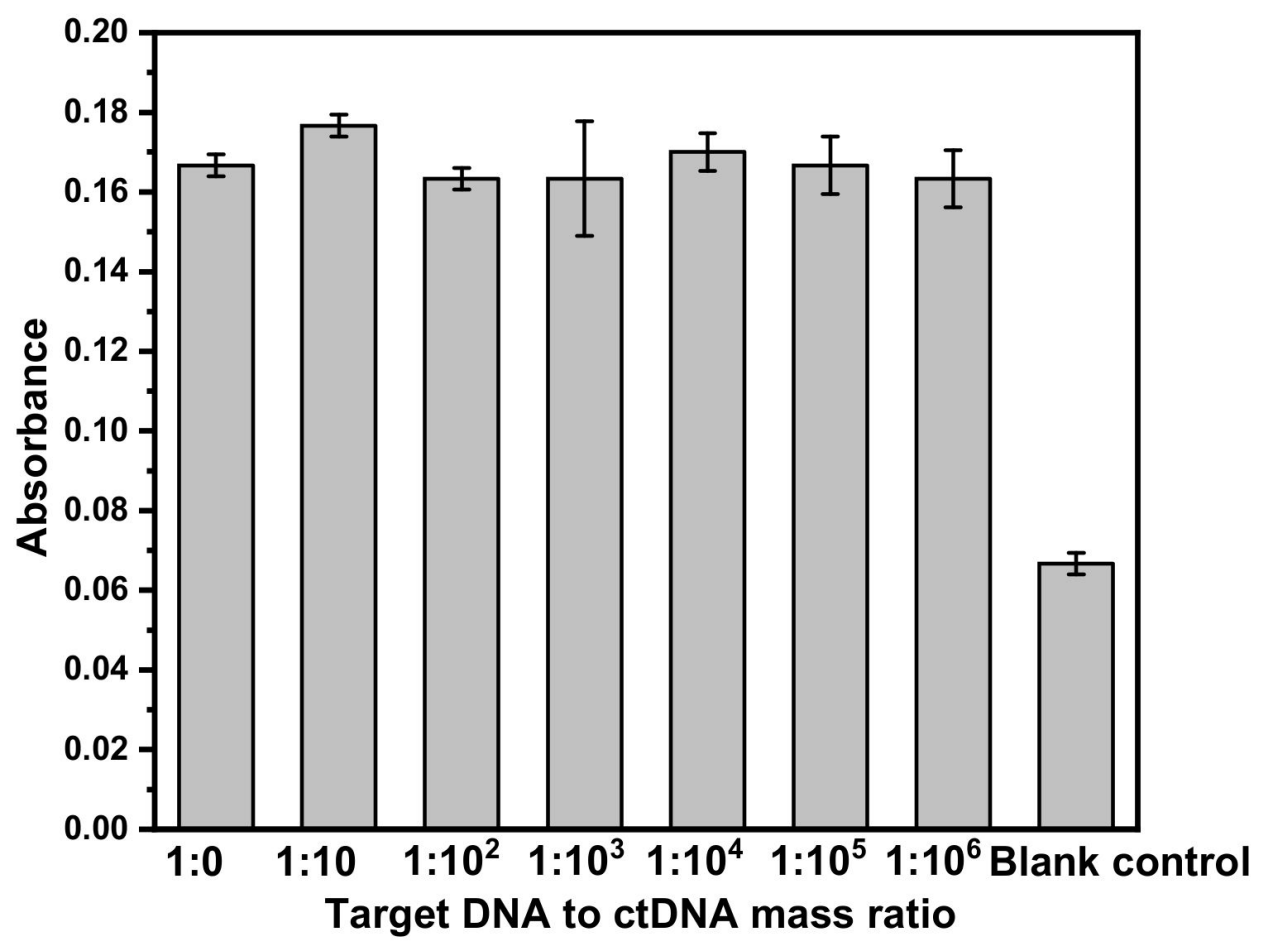
The absorbance signal of 1 pM target DNA in the presence of increasing amounts of ctDNA.

In order to demonstrate the potential in practical applications for DNA detection, we evaluated the selectivity in bulk genomic DNA. Calf thymus DNA (ctDNA) was used as non-target interference and an imitator for a complicated matrix at the same time. As shown in Figure 4, when the target was 1 pM, and the target DNA to genomic DNA ratio (in mass) from 1:0 to 1:10^6^, no significant change of the absorbance signals was observed. This result indicated that interference from the bulk genomic DNA was minimal, showing reasonable selectivity.

To further verify the possibility of the proposed method in real samples, spike recovery experiments were carried out for clinical application validation. Twenty oropharyngeal swab samples were acquired from healthy donors. The genomic DNAs were extracted from using a commercial kit from Roche according to the manufacturer’s instructions with the final volume 900 μL. Another genomic DNA from bacterium that causes lung disease, *Klebsiella pneumoniae* strains (>10^8^ CFU/mL), was also prepared with a final volume of 300 μL. A volume of 15 μL of target DNA with concentrations as indicated and 15 μL of extracted genomic DNA were added in the assay mixture with a final volume of 40 μL. Assays without the standard targets were carried out in parallel (Table 1). It was found that the percent recovery of the spike was 86.2% for 100 pM target and 100.0% for 1 pM target with the relative standard deviation (RSD) of 12.2% and 7.9%, with genomic DNA of healthy donors, respectively. The spike recovery was 93.1% for 100 pM target and 105.6% for 1 pM target with the RSD of 2.4% and 3.9% with genomic DNA of *Klebsiella pneumoniae*, respectively. These preliminary results showed that the proposed method has the feasibility for application with biological samples.

## 4. Conclusions

In summary, we constructed an ultra-sensitive method for *Mycobacterium tuberculosis*-related DNA detection with the dark-field microscopic imaging of AuNPs counting strategy and RCA. This proposed method is performed on an entry-level dark-field microscope setup with only a 6 μL detection volume. About 10 fM *Mycobacterium tuberculosis*-specific DNA target can still be differentiated from the blank. No significant change of the absorbance signals was observed when the target DNA to genomic DNA ratio (in mass) from 1:0 to 1:10^6^, showing reasonable selectivity. The spike recovery results in genomic DNA from human and *Klebsiella pneumoniae* suggested that the proposed method has feasibility in applications with biological samples. Due to the necessity for sophisticated instrumentation and nanoparticle monodispersity being significantly reduced, the proposed method showed promising potential for achieving high sensitivity in *Mycobacterium tuberculosis* clinical diagnosis and pathogen detection.

## Figures and Tables

**Table 1:** Spike recovery of 100 pM and 1 pM in genomic DNAs of human and *Klebsiella pneumoniae*, respectively (*n* = 2).

Target	100 pM	1 pM
Genomic DNA of healthy donors	86.2% ± 12.2%	100.0% ± 7.9%
Genomic DNA of *Klebsiella pneumoniae*	93.1% ± 2.4%	105.6% ± 3.9%

## Data Availability

Not applicable.

## Supplementary Materials

The following supporting information can be downloaded at: https://www.mdpi.com/article/10.3390/bios12070448/s1, Figure S1: (a) The DNA-HS concentrations as 0.1 μM, 0.25 μM and 1 μM on a certain amount AuNPs; (b) Dark-field images of AuNPs of the RCA reaction time; (c) Dark-field images of AuNPs for hybridization temperature; Table S1: Sequences of oligonucleotides used in Scheme 1; Table S2: Table S2 Comparison of the proposed method with published works with or without gold nanoparticles in recent years [36,37,38,39,40,41].
