# Supplementary material for: Nucleic Acids Detection for Mycobacterium tuberculosis Based on Gold Nanoparticles Counting and Rolling-Circle Amplification
Source: Biosensors (Basel). 2022 Jun 23;12(7):448. doi: 10.3390/bios12070448 (PMC9312627; doi:10.3390/bios12070448)
Supplement: Supplementary file 1 [file biosensors-12-00448-s001.zip › biosensors-1661908-supplementary.pdf]

Supporting Information

Nucleic Acids Detection for *Mycobacterium tuberculosis* Based on Gold Nanoparticles Counting and Rolling-Circle Amplification

Xiaojing Pei [1,*], Hu Hong [2], Sitong Liu [1] and Na Li [2,*]

[1]College of Chemistry and Materials Engineering and Institute of Cosmetic Regulatory Science, Beijing Tecnology and Business University, Beijing 100048, China, pxj@btbu.edu.cn

[2]Beijing National Laboratory for Molecular Sciences (BNLMS), Key Laboratory of Bioorganic Chemistry and Molecular Engineering of Ministry of Education, Institute of Analytical Chemistry, College of Chemistry and Molecular Engineering, Peking University, Beijing, 100871, China, lina@pku.edu.cn

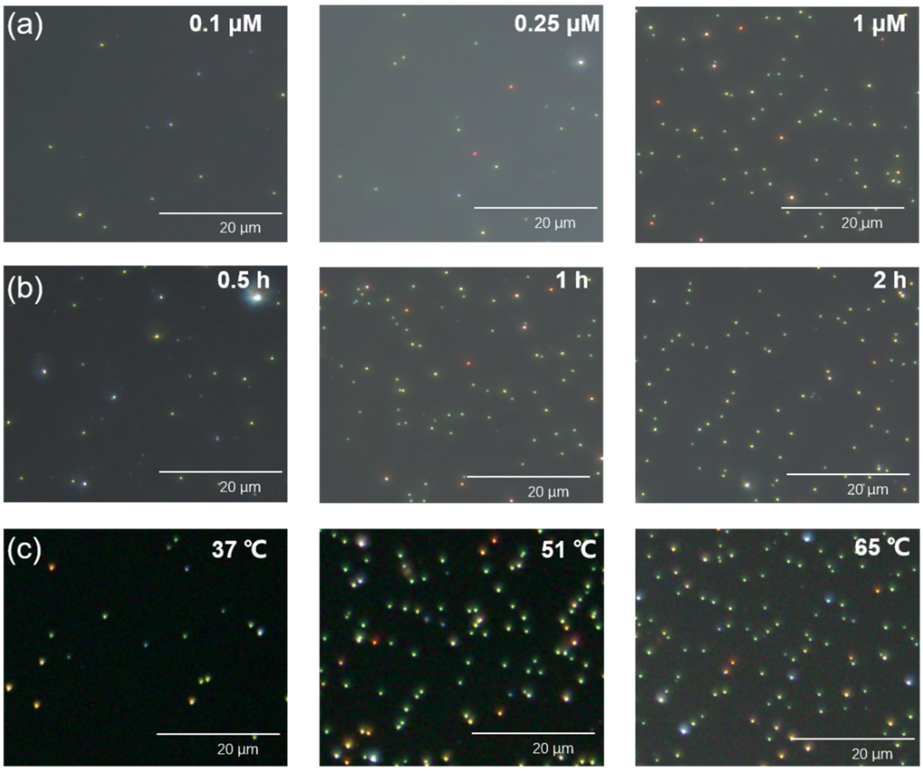

Figure S1. (a) The DNA-HS concentrations as 0.1 μM, 0.25 μM and 1 μM on a certain amount AuNPs; (b) Dark-field images of AuNPs of the RCA reaction time; (c) Dark-field images of AuNPs for hybridization temperature.

Table S1. Sequences of oligonucleotides used in **Scheme 1**.

**Name**	**Sequences**	**Description**
Target	CTCACCTATGTGTCGACCTGGGCAGGGT	
Capture DNA	AAAAAAAAAA GACCCGTTTCAAGATCC	5'biotin
Signaling DNA	GGCTCACCTCCTGCAGCTTCTGC A20	3'SH
Template	CGACACATAGGTGTAGGCTCACCTCCTGCAGCTTCTGC AATTCATCCGCGCCTCGCGCGGATGAAAA GACCCGTTTCAAGATCCAAACCCTGCCCAGGT*	5'P
Enzyme digestion product**	TTGCAGAAGCTGCAGGAGGTGAGCCTA CTCACCTATGTGTCGACCTGGGCAGGGT TTGGATCTTGAAACGGGTCTTTT	

*Purple indicates the recognition site of endonuclease; **For the convenience of readers, the enzyme digestion product is listed for reading, not the purchased sequence.

Table S2. Comparison of the proposed method with published works with or without gold nanoparticles in recent years.

Methods	Gold nanomaterials	LOD	Linear range	Real sample	References
AuNPs Counting	Signal readout	10 fM to 10 pM	10 fM	Spike recovery from bacterial lysates	**This work**
Paper Hybrid Device Using a Thermometer	AuNPs catalyze the oxidization reaction of TMB in the presence of H_2O_2	39 nM	100 nM ~ 50 μM	No	**[1]**
Nanocobalt and QDs	NO	24 pM	0.04-27 nM	Clinical MDR-TB strains	**[2]**
Bifunctionalized gold nanoparticles	chemiluminescent reagent and catalyst as signal reporters	48 fM	0.1 pM – 10 nM	Spike recovery from human serum samples	**[3]**
LAMP and CRISPR-Cas12b	NO	1.3 copies/ μL	NA	clinical samples	**[4]**
Paired dCas9	NO	30 aM	NA	clinical samples	**[5]**
CRISPR-Cas13a	NO	1×10^2copies/ μL (D94G)	NA	75 clinical samples	**[6]**

References

1. Wan Zhou, J. S., XiuJun Li, Low-Cost Quantitative Photothermal Genetic Detection of Pathogens on a Paper Hybrid Device Using a Thermometer. *Anal. Chem* **2020,** *92*, 14830-14837.

2. Ou Hu, Z. L., Qidi He, Yanli Tong, Yaoju Tan, Zuanguang Chen, Fluorescence Biosensor for One-Step Simultaneous Detection of Mycobacterium tuberculosis Multidrug-Resistant Genes Using nanoCoTPyP and Double Quantum Dots. *Anal. Chem* **2022**, 10.1021/acs.analchem.2c00723.

3. Lingfeng Gao, X.H.; Li Ju,Xiaoying Liu,Fang Li,Hua Cui, A label-free method for the detection of specific DNA sequences using gold nanoparticles bifunctionalized with a chemiluminescent reagent and a catalyst as signal reporters. *Anal. Bioanal. Chem.* **2016,** *408*, 8747–8754.

4. Wang, Y. TB-QUICK: CRISPR-Cas12b-assisted rapid and sensitive detection of Mycobacterium tuberculosis. *J. Infect* **2021,** *83*, 54-60.

5. Yihao Zhang , Y. W., Luze Xu , Chunbo Lou , Qi Ouyang , Long Qian, Paired dCas9 design as a nucleic acid detection platform for pathogenic strains. *Methods* **2021**, 10.1016/j.ymeth.2021.06.003.

6. Long, Q., A Highly Sensitive and Specific Detection Method for Mycobacterium tuberculosis Fluoroquinolone Resistance Mutations Utilizing the CRISPR-Cas13a System. *Front. Microbiol.* **2022,** *13*, 847373.
